# Biopharmaceutical Profiling of Herpetrione: A BCS II Properties and P-gp Substrate Guiding Nanoparticle Design

**DOI:** 10.3390/pharmaceutics18091142

**Published:** 2026-09-10

**Authors:** Fang Wang, Xiang Deng, Yuwen Zhu, Xinyu Zong, Yazhong Ma, Hailong Yuan

**Affiliations:** Department of Pharmacy, Air Force Medical Center, PLA, Air Force Medical University, Beijing 100142, China; 13916294560@163.com (F.W.); dengxiang0112@163.com (X.D.); 19271042963@163.com (Y.Z.); zongxinyu0910@163.com (X.Z.)

**Keywords:** herpetrione, biopharmaceutics classification system, p-glycoprotein, solubility, permeability, nanoparticles

## Abstract

**Objectives:** Herpetrione (HPE) is a bioactive lignan recognized for its hepatoprotective properties; however, it exhibits limited oral bioavailability. This study aimed to classify HPE within the framework of the biopharmaceutics classification system (BCS) and to develop a nanoparticle formulation. **Methods:** To this end, a comprehensive investigation was conducted, encompassing computer prediction analysis, equilibrium solubility measurements across the gastrointestinal pH range, Caco-2 bidirectional transportation, in situ single-pass intestinal perfusion (SPIP), and molecular docking with P-glycoprotein (P-gp). **Results:** In silico analyses suggested that HPE possesses low solubility and low permeability characteristics. Experimental assays revealed pH-dependent solubility and inherently low aqueous dissolution. Unlike the computer prediction results, Caco-2 studies revealed moderate permeability but a high efflux ratio, suggestive of possible P-gp substrate activity for HPE, a finding further supported by molecular docking simulations. Conversely, SPIP studies demonstrated that the effective permeability (*P*_eff_) of jejunal intestinal segments exceeded the high-permeability threshold, thereby classifying HPE as a high-permeability drug. Based on these findings, HPE was classified as a BCS class II compound. To overcome its solubility-limited absorption, a nanoparticle was developed, resulting in a marked enhancement of both solubility and dissolution rates. **Conclusions:** These findings underscore the importance of integrating experimental biopharmaceutical evaluations with computational tools when designing delivery systems for natural products.

## 1. Introduction

Herpetrione (HPE) is a bioactive lignan derived from the seeds of *Herpetospermum caudigerum* Wall., a traditional Tibetan medicinal plant extensively utilized for the treatment of hepatobiliary disorders such as hepatitis and cholecystitis [1,2]. Pharmacological investigations have revealed that HPE possesses hepatoprotective, anti-hepatitis B, antioxidant, and anti-tumor properties [3,4]. Despite these promising pharmacological effects, the clinical application of HPE is severely restricted by its poor oral bioavailability. Pharmacokinetic studies have reported an absolute oral bioavailability of less than 12% in rats, with the limited systemic exposure primarily attributed to poor aqueous solubility and possible permeability challenges [5].

The biopharmaceutics classification system (BCS) divides drugs into four groups according to their water solubility and ability to permeate the intestines [6]. This framework has been adopted by regulatory authorities globally, including the FDA, EMA, and NMPA, to guide formulation development and support biowaiver decisions [7,8,9]. Accurate BCS classification is thus crucial for predicting oral absorption behavior and facilitating rational formulation design. However, for natural products, BCS classification often presents challenges due to their structural complexity and limited prior characterization.

The BCS delineates distinct formulation strategies for different classes of drugs. Conventional formulations are usually sufficient for BCS class I drugs. Conversely, for drugs classified as BCS class II, which have low solubility but high permeability, the primary aim is to enhance solubility [10,11]. Nanotechnology has become an effective approach to enhance the oral absorption of drugs with poor water solubility [12,13]. By reducing particle size to the nanometer scale, nanoparticles (NPs) increase surface area and saturation solubility, thereby enhancing the dissolution rate and oral bioavailability. For BCS class III drugs, which are highly soluble yet have low permeability, it is important to enhance their permeability. This involves using lipid-based delivery systems, permeation enhancers, and efflux transporter inhibitors [14,15]. To address the low solubility and permeability of BCS class IV drugs, it is necessary to employ approaches that address both limitations, often requiring combination strategies [16,17,18]. Consequently, the rational development of NP formulation guided by BCS classification offers significant potential to overcome challenges in the absorption of natural drugs.

Over the past decade, researchers have explored various formulation strategies for HPE, including nanosuspensions stabilized by different surfactants, amorphous nanoparticles, and self-assembled nanoparticle systems [19,20,21]. Nanosuspensions prepared by high-pressure homogenization significantly increased *AUC*_0-t_ and *C*_max_ by 2.49-fold and 2.45-fold, respectively, compared to coarse HPE. Subsequent studies demonstrated that a particle size reduction from 450 nm to 200 nm further improved oral bioavailability, confirming their size-dependent absorption behavior [5,22]. Although these methods have shown improved solubility and bioavailability in preclinical models, the fundamental biopharmaceutical properties of HPE remain poorly characterized. Given the growing interest in developing oral nano-formulations of HPE, an in-depth understanding of its BCS classification is essential for guiding formulation strategies. Furthermore, while in silico approaches provide rapid predictions of drug permeability, their accuracy when used for complex natural products necessitates experimental validation. This study, therefore, integrates computational predictions with comprehensive experimental evaluations, including multi-pH solubility determination, Caco-2 bidirectional transport assays, and in situ single-pass intestinal perfusion, to establish the BCS classification of HPE. Based on this classification, a NP formulation was developed and characterized to enhance its solubility. This comprehensive biopharmaceutics characterization is expected to establish a scientific foundation for the rational design of oral formulations of HPE and provide a reference for the BCS-guided development of structurally similar natural products.

## 2. Materials and Methods

### 2.1. Chemicals and Reagents

HPE was isolated and purified from the seeds of *Herpetospermum caudigerum* Wall. in the laboratory of Dr. Yuan. The detailed isolation procedure was as follows. Dried seeds were powdered and extracted with 80% aqueous ethanol under reflux. The resulting extract was concentrated and sequentially partitioned with petroleum ether and ethyl acetate. The ethyl acetate fraction was subjected to repeated column chromatography on silica gel, sephadex LH-20 (Merck, Darmstadt, Germany), and semi-preparative high-performance liquid chromatography (semi-HPLC, LC-20A, Shimadzu, Japan), to yield HPE with a purity exceeding 98%. The Millicell^®^ cell culture inserts, featuring a 0.4 µm pore size, were obtained from Merck Millipore Ltd. (Darmstadt, Germany). Methanol, acetonitrile, and ethanol came from Fisher Scientific (Waltham, MA, USA). All other chemicals were of analytical grade.

The Caco-2 cell line (Caco-2) was bought from the Institute of Chinese Academy of Medical Sciences (Beijing, China). High-glucose Dulbecco’s Modified Eagle Medium (DMEM) was purchased from Procell (Wuhan, China). Penicillin-streptomycin double-antibody solution and fetal bovine serum were purchased from Gibco (Gran Island, NY, USA).

Male Sprague Dawley (SD) rats, with a weight of 200 ± 10 g, were acquired from SPF Biotechnology Co., Ltd. (Beijing, China). The Air Force Medical Center Animal Care and Use Committee approved the experimental procedures (No. 2023-57-PJ01), which adhered to the NIH Guidelines for animal care and use in experiments.

### 2.2. In Silico Prediction of Physicochemical Properties

The physicochemical properties of HPE were predicted utilizing three freely accessible computational tools, including ALOGPS 2.1 (https://vcclab.org/lab/alogps/, accessed on 22 November 2024), ADMETlab 3.0 (https://admetmesh.scbdd.com/, accessed on 22 November 2024), and SwissADME (http://www.swissadme.ch/, accessed on 22 November 2024) [23,24,25]. The predicted parameters encompassed molecular weight (MW), n-octanol/water partition coefficient (log *P*), the n-octanol/water distribution coefficient (log *D*), topological polar surface area (TPSA), hydrogen bond acceptors (HBAs), hydrogen bond donors (HBDs), rotatable bonds (RBs), and aqueous solubility (log *C*_s_). Additionally, permeability-related parameters, such as Caco-2 permeability, human intestinal absorption (HIA), and P-glycoprotein (P-gp) substrate status were obtained.

### 2.3. Solubility Determination

The pH-dependent solubility profile of HPE was determined using the shake flask method [26]. Water and a series of phosphate buffered saline (PBS) solutions with varying pH levels (pH 1.2, 2.0, 4.5, 5.8, 6.8, 7.4, and 8.0) were prepared. In brief, an extra amount of HPE was combined with 1.0 mL of buffer media and subjected to 30 min of ultrasonication. The mixture was then placed in a constant-temperature air shaker set at (37 ± 0.5) °C and agitated at 110 r·min^−1^ for 24 h. Once equilibrated, the samples underwent centrifugation at 14,000 r·min^−1^ for 15 min, filtered through 0.22 μm filters, diluted with methanol, and quantified using a previously developed and validated chromatographic method [22].

To classify HPE according to the BCS criteria, the dose number (*D*_0_) was calculated based on experimentally determined solubility data. *D*_0_ is a critical parameter that elucidates the relationship between drug solubility and oral absorption, defined by Equation (1) [6,27].

(1) D0=M0/V0Cs

where *M*_0_ represents the highest single oral dose of the drug (mg), *V*_0_ denotes the standard volume of water used to dissolve the drug, typically 250 mL, and *C*_s_ is the minimum solubility of the drug at (37 ± 0.5) °C across various pH media (g·L^−1^). According to the classical BCS theory, a *D*_0_ value of less than 1 indicates high solubility, whereas a *D*_0_ value equal to or greater than 1 indicates low solubility.

### 2.4. Partition Coefficient and Distribution Coefficient Determination

The n-octanol/water system was utilized in the shake-flask method. [28,29]. Sodium phosphate buffer at pH 7.4 was used for log *D* determination. Prior to the experiment, n-octanol and water were mutually saturated for 24 h at room temperature. In brief, 2.0 mg of HPE was added to 5 mL of water-saturated n-octanol. Following ultrasound treatment for 30 min, the solution was cooled to room temperature, diluted, and the initial concentration of HPE in the octanol phase was measured. Two milliliters of each solution were combined with two milliliters of an aqueous phase solution saturated with n-octanol. The mixture was subjected to ultrasonic treatment for 30 min and subsequently placed in a constant-air-temperature oscillator set at (37 ± 0.5) °C, with shaking at 110 r·min^−1^ for 24 h. Following this, the samples were centrifuged for 15 min at 14,000 r·min^−1^. The n-octanol phase was carefully collected from the upper layer, and the concentration of HPE in the aqueous phase was quantified using HPLC [30]. Log *P*/log *D* values were calculated using the total quantity of substance present in both phases according to Equations (2) and (3) [31].

(2) P=C0V0−CVCV

(3) $$logP={log}_{10}(P)$$

where *C*_0_ represents the initial concentration of HPE in water-saturated n-octanol (μg·mL^−1^), and *V*_0_ denotes the volume of water-saturated octanol (2 mL). *C* represents the ultimate concentration of HPE in the balanced aqueous phase (μg·mL^−1^), and *V* represents the volume of this phase (2 mL).

### 2.5. Permeability Investigation

#### 2.5.1. Cell Culture and Monolayer Formation

Caco-2 cells were grown in DMEM supplemented with 10% (*v*/*v*) fetal bovine serum, 1% nonessential amino acids, and 1% (*v*/*v*) penicillin-streptomycin double-antibody solution, kept at (37 ± 0.5) °C in an atmosphere containing 5% CO_2_ with 90% humidity. The cells were cultured on Transwell inserts with a 12 mm diameter and 0.4 μm pore size at a density of 1.0 × 10^5^ cells·cm^−2^ and grown for 21 days to achieve confluent monolayers. The integrity of the monolayer was evaluated by measuring transepithelial electrical resistance (TEER) using a cell resistance meter (Jing gong hong tai, Beijing, China). Only monolayers with TEER values higher than 500 Ω·cm^2^ were employed in permeability experiments.

#### 2.5.2. Cytotoxicity Assay

Cell viability was evaluated utilizing the CCK-8 assay. Caco-2 cells were specifically seeded into 96-well plates at a density of 5 × 10^4^ cells·cm^−2^ and kept under the previously described conditions until differentiation was achieved. The cells, once differentiated, were subjected to various concentrations of HPE for 4 h. Following this, 10% CCK-8 was introduced to each well and incubated at 37 °C for 3 h. Absorbance was recorded at the wavelength of 450 nm with a microplate reader. Relative cell viability was determined by normalization to the 100% viability benchmark set by the DMEM.

#### 2.5.3. Bidirectional Transport Study

To determine HPE bioavailability, its permeability through fully differentiated Caco-2 cell monolayers was evaluated. Bidirectional transport studies were performed on 21-day-old Caco-2 monolayers exhibiting stable TEER values. The monolayers were pre-incubated with DMEM buffer at 37 °C for 30 min. Based on the cytotoxicity findings, HPE was prepared in DMEM at a concentration of 125 μg·mL^−1^ for the transport experiments. For apical-to-basolateral (AP→BL) transport, the drug solution was placed in the AP, while the BL compartment received fresh DMEM. Conversely, for basolateral-to-apical (BL→AP) transport, the drug solution was put into the BL, with fresh DMEM introduced into the AP compartment. The plates were incubated at (37 ± 0.5) °C with gentle agitation. Samples (200 μL) were taken from the receiver compartment at intervals of 30, 60, 120, and 240 min and were promptly replaced with the same volume of pre-warmed fresh DMEM. All samples were stored at −20 °C until analysis. Using LC-MS/MS, the concentrations in the samples were measured, and the apparent permeability coefficient (*P*_app_) was calculated using Equation (4) [32].

(4) $$P_{\mathrm{app}}=(dC/dt)/(AC_{0})$$

where *d*C/*dt* represents the constant flux (μg·s^−1^), *A* stands for the surface area of the membrane (1.12 cm^2^ for 12 mm inserts), and *C*_0_ refers to the starting concentration in the donor section (μg·mL^−1^).

The efflux ratio (*ER*) was calculated as shown in Equation (5) [33].

(5) $$ER=P_{app(BL\to AP)}/P_{app(AP\to BL)}$$

According to the literature [34], an ER value exceeding 2 suggests the participation of active efflux transporters like P-gp or other ATP-binding cassette (ABC) transporters. Conversely, an ER value less than or equal to 2 suggests that active efflux is not significant.

#### 2.5.4. LC-MS/MS Analysis

Chromatographic separation was performed on an ACQUITY UPLC BEH C18 column (2.1 × 50 mm, 1.7 μm, Waters, MA, USA) maintained at 35 °C. The mobile phase consisted of (A) 0.1% formic acid in water and (B) acetonitrile, with gradient elution at a flow rate of 0.2 mL/min. The gradient mode was 10~30% of phase A for 3 min, 40~95% for 2 min, and 10% for 1 min. The injection volume was 5 μL. Detection was carried out on a triple quadrupole mass spectrometer equipped with an electrospray ionization (ESI) source operating in positive ion mode. HPE was quantified using multiple reaction monitoring (MRM).

### 2.6. In Situ Single-Pass Intestinal Perfusion

#### 2.6.1. Surgical Procedure

Based on earlier studies and published articles, the experimental procedure for the in situ single-pass intestinal perfusion (SPIP) studies was carried out [35]. Male SD rats were deprived of food for 12 h overnight but had unlimited access to water. Anesthesia was induced via intraperitoneal injection of 2.5% tribromoethanol (10 mL·kg^−1^). An incision approximately 4 cm in length was made along the abdominal midline. The intestinal segments selected for perfusion comprised the duodenum (located 2 to 12 cm away from the pylorus), the jejunum (5 to 15 cm beyond the ligament of treitz), the ileum (10 cm before the cecum), and the colon (2 to 10 cm proximal to the anus). Each segment, approximately 10 cm in length, was cannulated with silicone tubing at both ends and secured using sterilized surgical sutures. To keep the moisture levels stable during the experiment, the intestinal segments were enveloped in gauze moistened with normal saline, and body temperature was sustained at 37 °C via an infrared lamp.

#### 2.6.2. Perfusion Protocol

The intestinal contents were softly washed with normal saline preheated to 37 °C. The segments were balanced with blank Krebs-Ringer buffer at pH 6.8, flowing at 0.2 mL·min^−1^ for 15 min. Subsequently, a perfusion solution containing HPE at a concentration of 20 μg·mL^−1^ was introduced at the same flow rate. After a 30 min equilibration period, samples were collected from the perfusate at 15 min intervals over a 120 min period. Pre-weighed vials were utilized to collect the outflow solution, with the weights of both inlet and outlet vials recorded to correct for water flux. All samples were preserved at −20 °C until analysis.

#### 2.6.3. Sample Analysis

The concentration of the drug in perfusate samples was quantified using HPLC. The effective permeability coefficient (*P*_eff_) was determined using Equation (6) [36].

(6) $$P_{eff}=-Qln(\frac{C_{out}}{C_{in}})/2\pi rL$$

where *Q* stands for the perfusion flow rate (mL·s^−1^), *C*_in_ and *C*_out_ indicate the drug concentrations at the inlet and outlet (μg·mL^−1^), *r* denotes the radius of the intestinal segment (cm), and *L* is the length of the segment being perfused (cm). Following perfusion, the rats were euthanized, and the length and inner diameter of each perfused intestinal segment were measured.

### 2.7. Molecular Docking Analysis

Molecular docking was conducted to predict the binding mode of HPE with human P-gp by AutoDock Vina 1.2.2. The three-dimensional (3D) structure of HPE was retrieved from the PubChem database (CID: 16037396), and quercetin (CID: 5280343) was used as a positive control. The Protein Data Bank was used to download the crystal structure of human P-gp (PDB ID: 6C0V; resolution: 3.4 Å). Prior to molecular docking, the original ligand of the protein was obtained as an internal control. The protein structure was prepared by removing the original ligands and crystallographic water molecules, followed by the addition of polar hydrogen atoms. A grid box of 30 Å × 30 Å × 30 Å with a grid spacing of 0.05 nm (0.5 Å) was centered on the putative substrate-binding pocket to accommodate flexible ligand movement. Among the generated binding poses, the conformation with the most favorable binding affinity was visualized using PyMOL software (version 2.5.7).

### 2.8. Preparation of HPE-NPs

Preparation of the NPs was carried out using the solvent precipitation method with modifications [37]. HPE (100 mg) was dissolved in 1 mL of ethanol, serving as the organic phase, with hydroxypropyl methylcellulose (HPMC E15) employed as the stabilizer at a 4:1 weight ratio of drug to stabilizer. This organic phase was then added to 10 mL of an aqueous stabilizer solution, stirred at 400 r·min^−1^ for 20 s. The resulting nanosuspension underwent vacuum evaporation at 40 °C to remove ethanol.

### 2.9. Characterization of HPE-NPs

#### 2.9.1. Particle Size, Polydispersity Index, and Zeta Potential

Dynamic light scattering (DLS) with a Nanotrac Wave II analyzer (Microtrac Inc., Montgomeryville, PA, USA) was employed to measure the mean particle size and polydispersity index (PDI). The measurement of the Zeta potential was conducted with a Zeta potential analyzer (JS94K2, Shanghai Zhong chen Digital Technic Apparatus, Shanghai, China). All measurements were conducted in triplicate after appropriate dilution with deionized water.

#### 2.9.2. Morphological Observation

Scanning electron microscopy (SEM; S-4800, Hitachi Technologies, Tokyo, Japan) was employed to assess the morphological traits of the samples. The samples were mounted on a metal holder with double-sided conductive tape and coated with a thin layer of gold in a vacuum. Observations were carried out at an accelerating voltage of 5 kV.

#### 2.9.3. Drug Loading

For the determination of drug loading (DL), a 5 mL aliquot of the HPE-NP solution was subjected to freeze-drying and subsequently weighed. After dissolving the resulting powder in 25 mL of methanol, it was ultrasonically treated for 10 min. The solution was then centrifuged twice at 14,000 r·min^−1^ for 10 min, and the supernatant was collected for analysis.

#### 2.9.4. Encapsulation Efficiency

For the determination of encapsulation efficiency (EE), ultrafiltration and centrifugation techniques were employed. Specifically, 500 μL of HPE-NP formulation was placed in an ultrafiltration centrifuge tube (10 kDa). After centrifuging the samples at 14,000 r·min^−1^ for 60 min at 4 °C, the filtrate was diluted with methanol and measured. For quantifying the total amount of HPE in the HPE-NPs, 0.5 mL of the formulation was diluted with 5 mL of methanol and vortexed for 30 min to fully disrupt the NPs and release the encapsulated HPE. Before HPLC analysis, all samples were passed through a 0.22 μm membrane filter.

#### 2.9.5. X-Ray Diffraction

The crystalline state of HPE, its physical mixture, and HPE-NPs were characterized using a D8 ADVANCE diffractometer (Bruker, Karlsruhe, Germany) for X-ray diffraction (XRD), with Cu Kα radiation, operating at 40 kV and 40 mA, across a 2θ range from 3° to 60° at a scanning rate of 2°/min.

#### 2.9.6. Solubility and Dissolution Studies

The solubility equilibrium in water was determined following the procedure detailed in Section 2.3. In vitro dissolution studies were conducted using the dialysis bag method in PBS at pH 7.4, supplemented with 0.2% (*w*/*v*) Tween 80 to maintain sink conditions. Both HPE-NPs and raw HPE were placed inside dialysis bags with a molecular weight cut-off of 8~14 kDa and immersed in 50 mL of dissolution medium, maintained at (37 ± 0.5) °C, with stirring at 100 r·min^−1^. At predetermined time intervals (0.5, 1, 2, 4, 8, 12, 24, 36, and 48 h), 1 mL of each sample was withdrawn and replaced with a fresh medium. The concentration of HPE was determined by using HPLC. The release mechanism was elucidated by analyzing the dissolution data with zero-order, first-order, Higuchi, and Ritger-Peppas kinetic models.

### 2.10. Statistical Analysis

Results are expressed as mean ± standard deviation (SD) from a minimum of three independent trials. Statistical evaluations were carried out using one-way analysis of variance (ANOVA) followed by Tukey’s post hoc test with GraphPad Prism 8.0. Statistical significance was considered for *p* values smaller than 0.05.

## 3. Results and Discussion

### 3.1. Predicted Physicochemical and ADME Properties

**Figure 1:**
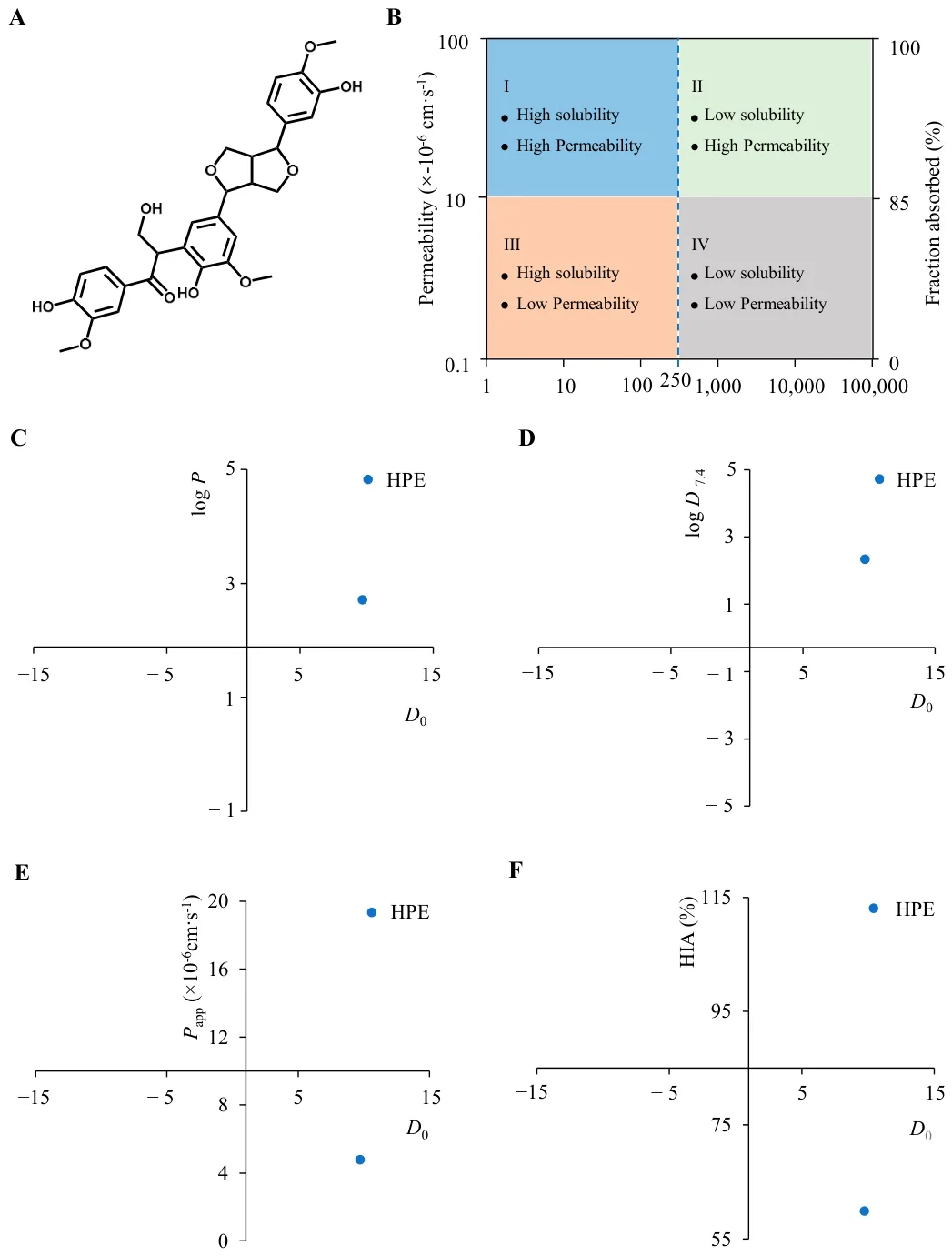
BCS classification of drug based on software prediction values. (**A**) Molecular structures of HPE; (**B**) The schematic diagram of BCS; (**C**) BCS classification based on *D*_0_ and log *p* value; (**D**) BCS classification based on *D*_0_ and log *D*_7.4_ value; (**E**) BCS classification based on *D*_0_ and *P*_app_ value; (**F**) BCS classification based on *D*_0_ and HIA value. I: BCS class I; II: BCS class II; III: BCS class III; IV: BCS class IV; *D*_0_: dose number; log *P*: the n-octanol/water partition coefficient; log *D*_7.4_: the n-octanol/water distribution coefficient; *P*_app_: apparent permeability coefficient; and HIA: human intestinal absorption.

All parameters related to physicochemical and biopharmaceutical properties, as predicted by various computational algorithms, are detailed in Table 1. The structure for this compound can be seen in Figure 1A. The MW of HPE slightly exceeds the Lipinski threshold yet remains within the acceptable range for oral drug development [38]. The predicted log *p* values demonstrated good consistency across platforms, with a consensus value of 2.65 ± 0.22. These values suggest moderate lipophilicity, which is generally favorable for passive membrane permeability [39]. Furthermore, the active compound met the criteria for extended and beyond Rule of Five (eRo5, bRo5) and the 3/75 rule of Pfizer [40,41,42]. Its predicted solubility (log *C*_s_) ranged from −4.89 to −4.46, equating to 7.12 to 19.23 μg·mL^−1^. The maximum human dose of HPE used for *D*_0_ calculation was derived from the traditional use of *Herpetospermum caudigerum* seeds. According to the Chinese Pharmacopoeia and relevant clinical practice, the maximum daily dose of the seeds is 6 g [43]. Based on the content of HPE in the seeds (5.0 mg·g^−1^) [44], the corresponding maximum daily dose of HPE was calculated as 30 mg. This calculation method follows the approach established by Fong et al. for the provisional BCS classification of Chinese herbal medicine markers, in which the daily dose of the herbal material is multiplied by the marker compound content to estimate the upper limit of exposure [45,46]. The calculated *D*_0_ from all predicted solubility values exceeded 1, consistently predicting low solubility according to BCS criteria.

The predicted Caco-2 permeability was −5.32 log units, corresponding to a *P*_app_ value of 4.79 × 10^−6^ cm·s^−1^, within the range associated with moderate passive diffusion. Computational prediction further indicated a 59.9% probability of moderate human intestinal absorption. In contrast, the compound was classified as having low gastrointestinal absorption by SwissADME. Both platforms consistently predicted that it is not a substrate for P-gp. The predicted log *P*, log *D*, *P*_app_, and HIA values were compared with that of the high permeability standard (Metoprolol, log *P* ≥ 1.88, log *D*_7.4_ = −0.287, *P*_app_ ≥ 10 × 10^−6^ cm·s^−1^, and HIA ≥ 85%) [32,47,48,49]. Collectively, these in silico assessments point toward low aqueous solubility and low-to-moderate permeability, features consistent with BCS class II or IV (Figure 1B–F). Given the known limitations of computational ADMET models, particularly in capturing complex physiological interactions, further experimental validation was performed using both Caco-2 cell monolayers and in situ SPIP in rats to rigorously confirm the BCS classification.

### 3.2. Solubility Behavior and Dose Number Analysis

**Figure 2:**
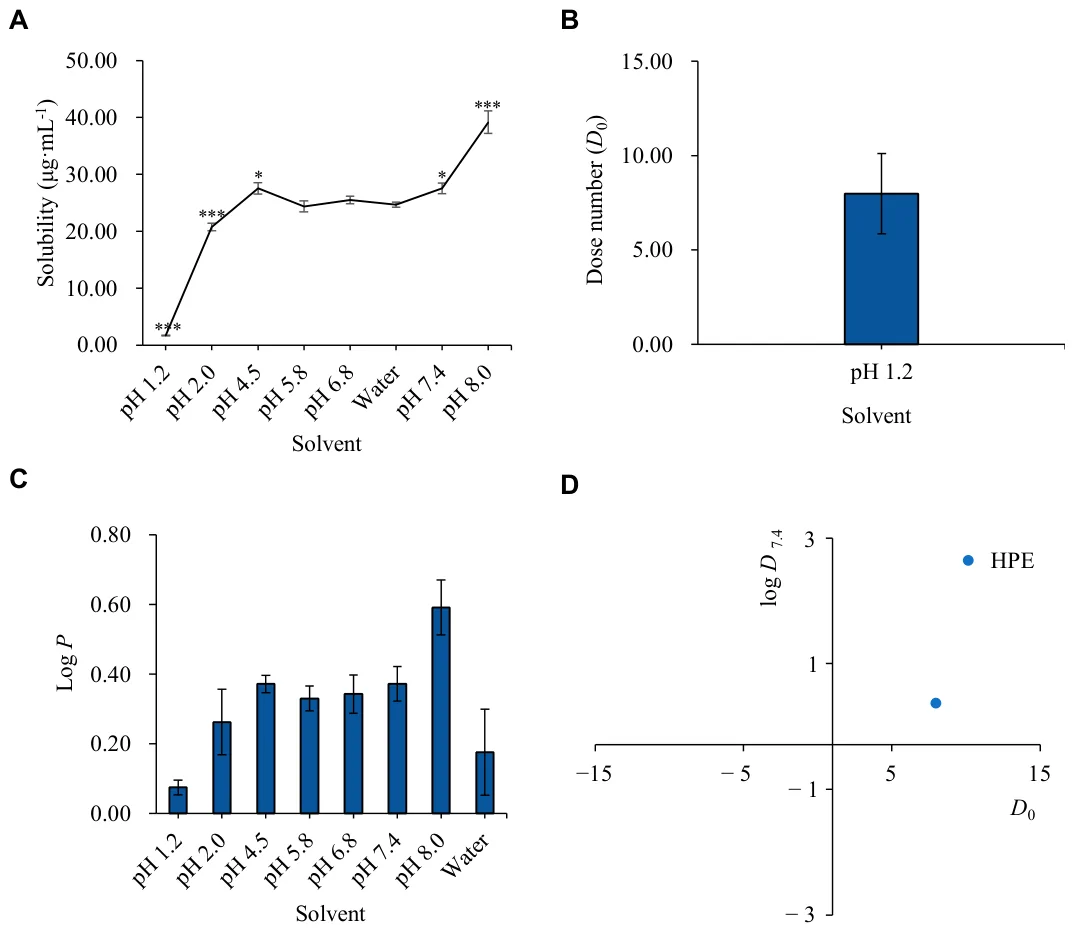
Biopharmaceutics characterization of HPE: solubility, *D*_0_, pH-dependent lipophilicity, and BCS classification. (**A**) Equilibrium solubility of HPE in different solvents (37 °C); (**B**) The *D*_0_ of HPE in pH 1.2 buffer solutions (48 h); (**C**) The log *P* of HPE in different buffers; (**D**) BCS classification based on experimental *D*_0_ and log *D*_7.4_ value. Data are presented as mean ± SD (*n* = 3). Statistical significance of differences solubility (vs. water), * *p* < 0.05, *** *p* < 0.001. *D*_0_: dose number; log *P*: the n-octanol/water partition coefficient; log *D*_7.4_: the n-octanol/water distribution coefficient.

The solubility of HPE in different buffer solutions at 37 °C iwas illustrated in Figure 2A. HPE demonstrates a distinct pH-dependent solubility profile, with the lowest solubility observed at pH 1.2 (1.67 ± 0.06 μg·mL^−1^) and the highest at pH 8.0 (39.17 ± 1.94 μg·mL^−1^). Its solubilityThere was a had significant difference betweenin its solubility in a buffer with different pH levels compared withand that in water. Although the solubility of HPE is low at the gastric fluid pH of 1.2, this does not pose a critical absorption barrier; as the primary absorption site, the small intestine provides a significantly favorable microenvironment that ensures a sufficient dissolved drug concentration for permeation.

The *D*_0_ value was calculated using the minimum solubility, which was observed at pH 1.2. The time-course experiment confirmed that a true thermodynamic equilibrium was reached at 48 h. The equilibrium solubility at pH 1.2 was determined to be (15.69 ± 3.65 μg·mL^−1^), yielding a *D*_0_ of 7.98. which substantially exceeds the BCS threshold of 1 (Figure 2B), thereby classifying HPE as a low-solubility drug.

### 3.3. pH-Dependent Lipophilicity

Figure 2C illustrates the log *p* values of HPE in different media, ranging from 0.07 to 0.95 across the tested pH conditions. This range suggests a favorable balance between solubility and permeability, indicating that HPE possesses moderate lipophilicity conducive to passive transcellular diffusion [50]. Taking into account the differences in the dissociation states of the drug at various pH levels, we define the log *D* as the value of log *P* at pH 7.4. The experimental (*D*_0_, log *D*_7.4_) values point toward low aqueous solubility and high permeability, features consistent with BCS class II (Figure 2D).

### 3.4. Evaluation of Permeability Across Caco-2 Cell Monolayers

#### 3.4.1. Cytotoxicity Results

**Figure 3:**
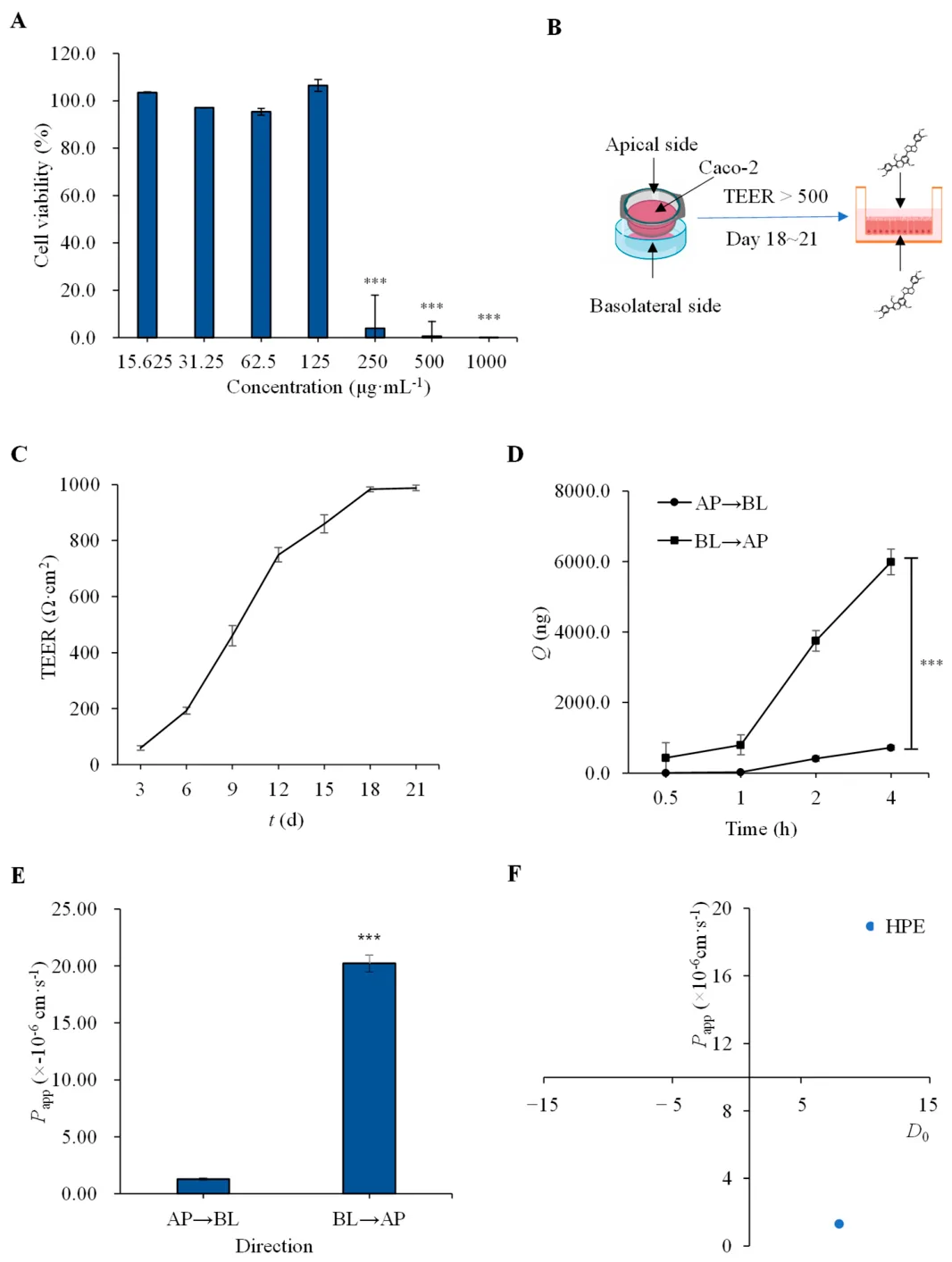
In vitro transport in Caco-2 cell monolayers. (**A**) Cytotoxicity of HPE on Caco-2 cells after 4 h incubation, relative cell viability was normalized to control cells; *** *p* < 0.001 significant vs. the negative control. (**B**) A schematic representation of Transwell experiments; (**C**) TEER values of Caco-2 cells; (**D**) Cumulative amount of HPE transports across Caco-2 cell monolayers over time in both directions; (**E**) The apparent permeability of the two-way transport of HPE; (**F**) BCS classification based on the experimental *D*_0_ and *P*_app_ value. Data are presented as mean ± SD (*n* = 3). Statistical significance of differences between transport directions (vs. AP→BL), *** *p* < 0.001. AP: apical side; BL: basolateral side. *D*_0_: dose number; *P*_app_: apparent permeability coefficient.

The CCK-8 assay results indicate that HPE does not show notable cytotoxic effects on Caco-2 cells at concentrations lower than 125 μg·mL^−1^ over a 4 h period (Figure 3A). Consequently, a concentration of 125 μg·mL^−1^ was selected for transport studies to ensure both cell viability and adequate analytical sensitivity.

#### 3.4.2. Bidirectional Transport of HPE in Caco-2 Cells

To investigate the transmembrane transport of HPE, a bidirectional transfer test was performed (Figure 3B). Figure 3C showed the membrane resistance values on different days, whereby all monolayers exhibited TEER ≥ 500 Ω·cm^−2^ after 21 days of culture. The cumulative transported amounts of HPE in both directions were increased with the exposure time (Figure 3D). By comparison, it was found that more HPE was excreted into the AP side than into the BL side (*p* < 0.001). According to established classification criteria, *P*_app_ values less than 1.0 × 10^−6^ cm·s^−1^ denote poor absorption, whereas values exceeding 1.0 × 10^−5^ cm·s^−1^ indicate high absorption [51]. The transport of HPE from the AP→BL in the Caco-2 cell model was moderate with a *P*_app_ value of (2.62 ± 0.21) × 10^−6^ cm·s^−1^, while the BL→AP value was (20.22 ± 0.74) × 10^−6^ cm·s^−1^ (Figure 3E). The ER was determined to be 7.75, significantly surpassing the threshold of 2, which strongly suggests the involvement of active efflux transporters. Collectively, these findings suggest that HPE is likely a substrate of P-gp or other ATP-binding cassette (ABC) transporters. [52]. Thus, in the static Caco-2 model, HPE appears as a compound with moderate intrinsic permeability but prominent efflux liability, a pattern that, if considered alone, would be compatible with low net permeability, features consistent with BCS class IV (Figure 3F). However, because Caco-2 monolayers lack the dynamic physiological environment (e.g., blood flow and sustained concentration gradient) that can overcome efflux in vivo, the definitive assignment of BCS permeability class relies on the in situ SPIP.

### 3.5. Evaluation of Regional Intestinal Permeability

**Figure 4:**
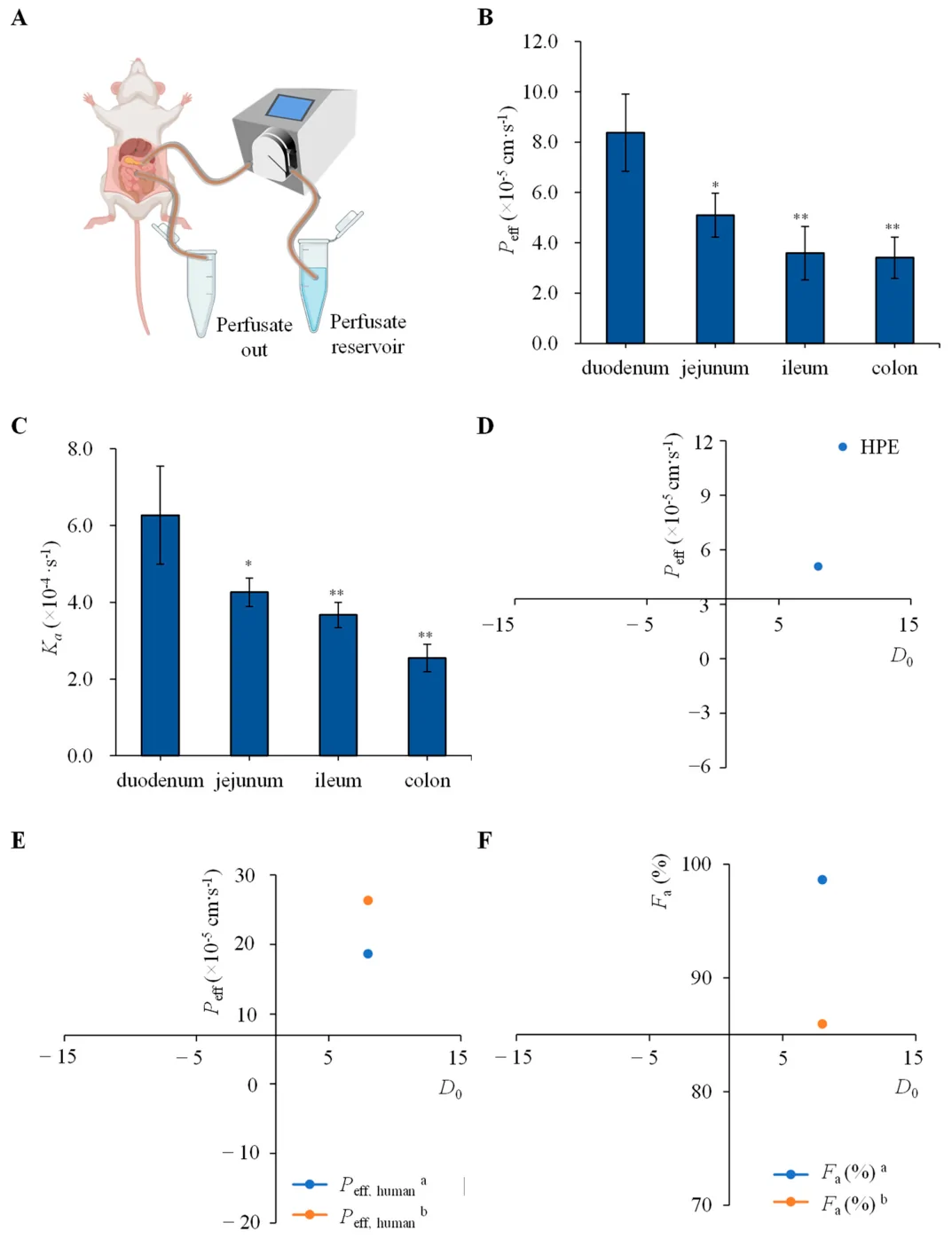
Regional intestinal absorption and BCS classification determined by in situ SPIP model. (**A**) Schematic diagram of the SPIP model. (**B**) The *P*_eff_ values of HPE in duodenum, jejunum, ileum, and colon segments. (**C**) The Ka values in the same intestinal segments. (**D**) BCS classification based on the experimental *D*_0_ and *P*_eff_ values in the jejunum. (**E**) BCS classification based on the experimental *D*_0_ and *P*_eff,human_ values in the jejunum. (**F**) BCS classification based on the experimental *D*_0_ and *F*a values in the jejunum. Data are presented as mean ± SD (*n* = 3). Significant differences (vs. duodenum), * *p* < 0.05, ** *p* < 0.01. *D*_0_: dose number; *P*_eff_: effective permeability coefficient; *P*_eff, human_: predicted human intestinal permeability; *F_a_*: fraction dose absorbed. a: The results were calculated using the methodology proposed by Fagerholm et al. [54]. b: The results were calculated using the methodology proposed by Zakeri-Milani et al. [55].

The SPIP model was used to further explore the intestinal permeability features in rats (Figure 4A). The evaluations of HPE’s absorption rate constant (Ka) and effective permeability coefficient (*P*_eff_) were conducted in various intestinal segments such as the duodenum, jejunum, ileum, and colon (Figure 4B,C). Both parameters demonstrated a pronounced segment-dependent decreasing trend from the proximal to the distal intestine: duodenum > jejunum > ileum > colon. Notably, the jejunal *P*_eff_ of HPE was higher than that of metoprolol (3.3 ×10^−5^ cm·s^−1^), the FDA-recommended high-permeability internal reference standard [53]. A comparison of the two, together with the quantitative estimation from the established correlation between rat and human intestinal permeability, yielded a predicted human fraction dose absorbed (*F_a_*) substantially exceeding 85% (Table 2). Notably, the predicted human intestinal permeability (*P*_eff, human_) from both the Fagerholm and Zakeri-Milani models further supported the high-permeability classification of HPE [54,55]. Collectively, these data unequivocally demonstrate that HPE possesses high permeability, confirming its classification as a BCS Class II drug (Figure 4D–F).

The markedly higher *P*_eff_ in the proximal intestine suggests that the absorption window for HPE is predominantly situated in the upper small intestine. Specifically, the *P*_eff_ in the duodenum was approximately 1.64 times greater than that in the jejunum, 2.33 times greater than that in the ileum, and 2.46 times greater than that in the colon. The coefficients of variation (CV) for *P*_eff_ ranged from 17.1% (jejunum) to 29.5% (ileum), while those for Ka ranged from 8.7% (jejunum) to 20.5% (duodenum). These values are within the acceptable range for in situ intestinal perfusion studies. The reduced permeability observed in the ileum and colon, along with the relatively greater variability in the ileum with the documented higher expression levels of P-gp and other ABC efflux transporters in these distal regions, align with the high efflux ratio observed in the Caco-2 assay [56]. In general, the intestinal absorption of orally administered drugs is modulated not only by passive permeability but also by active efflux mediated by ABC transporters, which pump a wide range of structurally diverse compounds back into the intestinal lumen. Among these, P-gp is the most extensively characterized apical efflux transporter in the human intestine, and its expression levels exhibit both regional and inter-individual variability [57,58]. Notably, this inference is further corroborated by our previous mechanistic study on HPE amorphous nanoparticles, in which verapamil, a classic P-gp inhibitor, significantly increased cellular HPE uptake [30], directly supporting the involvement of P-gp in HPE efflux.

### 3.6. Integrated BCS Classification

The BCS classification for HPE was identified through an extensive analysis of solubility and permeability information. In terms of solubility, HPE is categorized as a low-solubility compound, a classification consistently corroborated by both experimental measurements and in silico predictions. Regarding permeability, despite in silico predictions indicating low gastrointestinal absorption and a non-P-gp substrate status, experimental investigations revealed a contrasting profile. The Caco-2 model and in situ intestinal perfusion revealed moderate to high permeability and pointed to HPE as a possible substrate of efflux transporters. This discrepancy highlights the limitations of current computational models in accurately predicting transporter-mediated processes for complex natural products and underscores the necessity of experimental validation. Ultimately, HPE is classified as a BCS class II compound.

### 3.7. Predicted Binding Mode with P-gp

**Figure 5:**
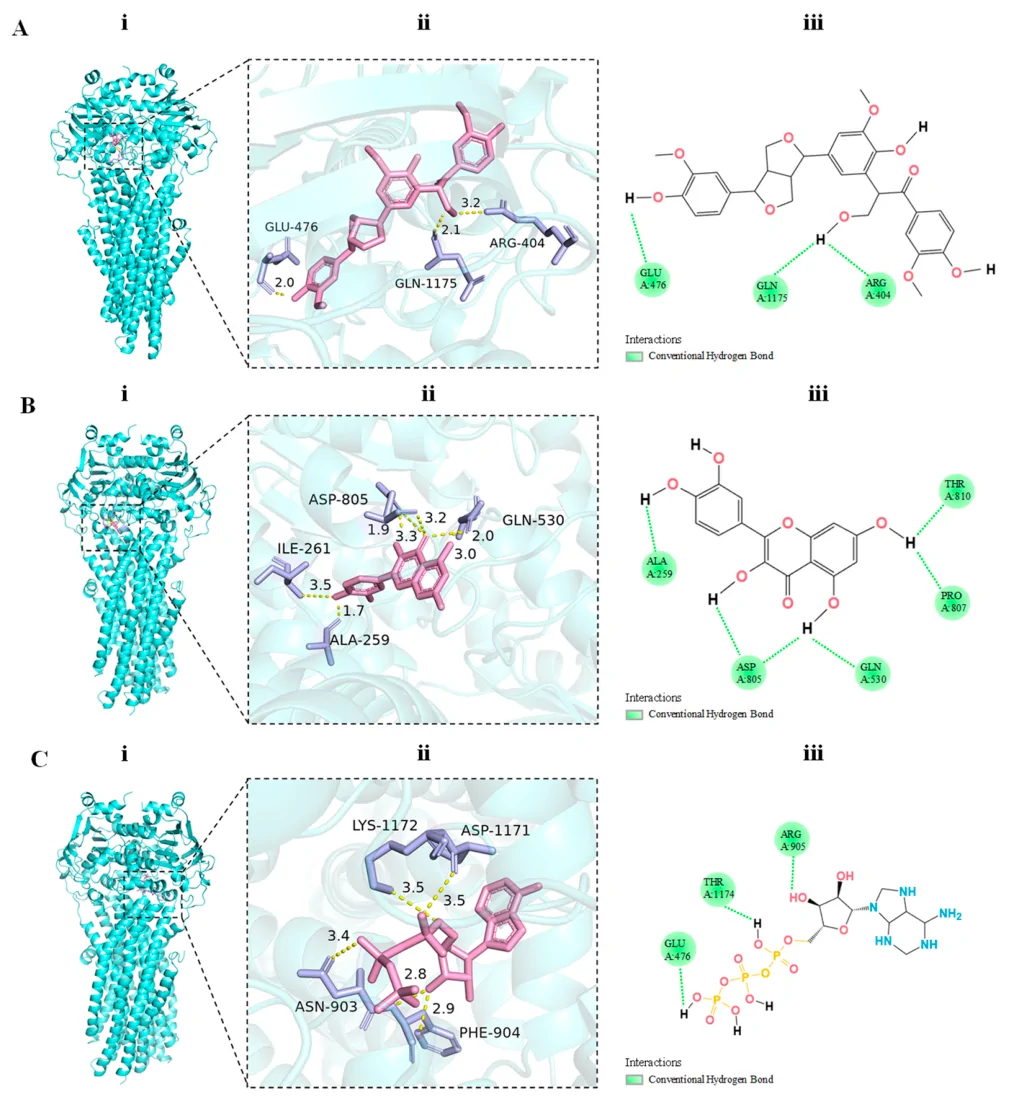
The binding interactions of ligands with their targets by using molecular docking. (**A**) Binding mode of HPE to 6C0V. (**B**) Binding mode of quercetin to 6C0V. (**C**) Binding mode of internal ligand to 6C0V. (**i**) Diagram of optimal molecular docking binding; (**ii**) PyMOL software display of the three-dimensional structures of the binding pockets; (**iii**) 2D interactions between compounds and their targets.

To further explore the molecular basis of this efflux behavior, molecular docking simulations were performed to evaluate the binding affinity and interaction mode of HPE within the P-gp substrate-binding pocket, using quercetin as a positive control and the original co-crystallized ligand for comparison. HPE showed a binding energy of −8.998 Kcal·mol^−1^, which was comparable to that of quercetin and the re-docked co-crystallized ligand (Table 3), and the optimal binding poses are visualized in Figure 5. The interaction of HPE with P-gp led to the formation of three hydrogen bonds with significant amino acid residues in the binding pocket, namely GLU476 (2.0 Å), GLN1175 (2.1 Å), and ARG404 (3.2 Å) (Figure 5A). The positive control (quercetin) established six hydrogen bonds with the surrounding residues, consistent with its known P-gp modulating activity (Figure 5B). The co-crystallized ligand was successfully re-docked, replicating the original binding mode (Figure 5C). These results indicate that HPE binds directly to the substrate-binding pocket of P-gp through favorable interactions, offering a structural basis for the high efflux ratio observed in the Caco-2 transport assay.

### 3.8. In Vitro Characterization of HPE-NPs

#### 3.8.1. Size Distribution and Surface Charge

**Figure 6:**
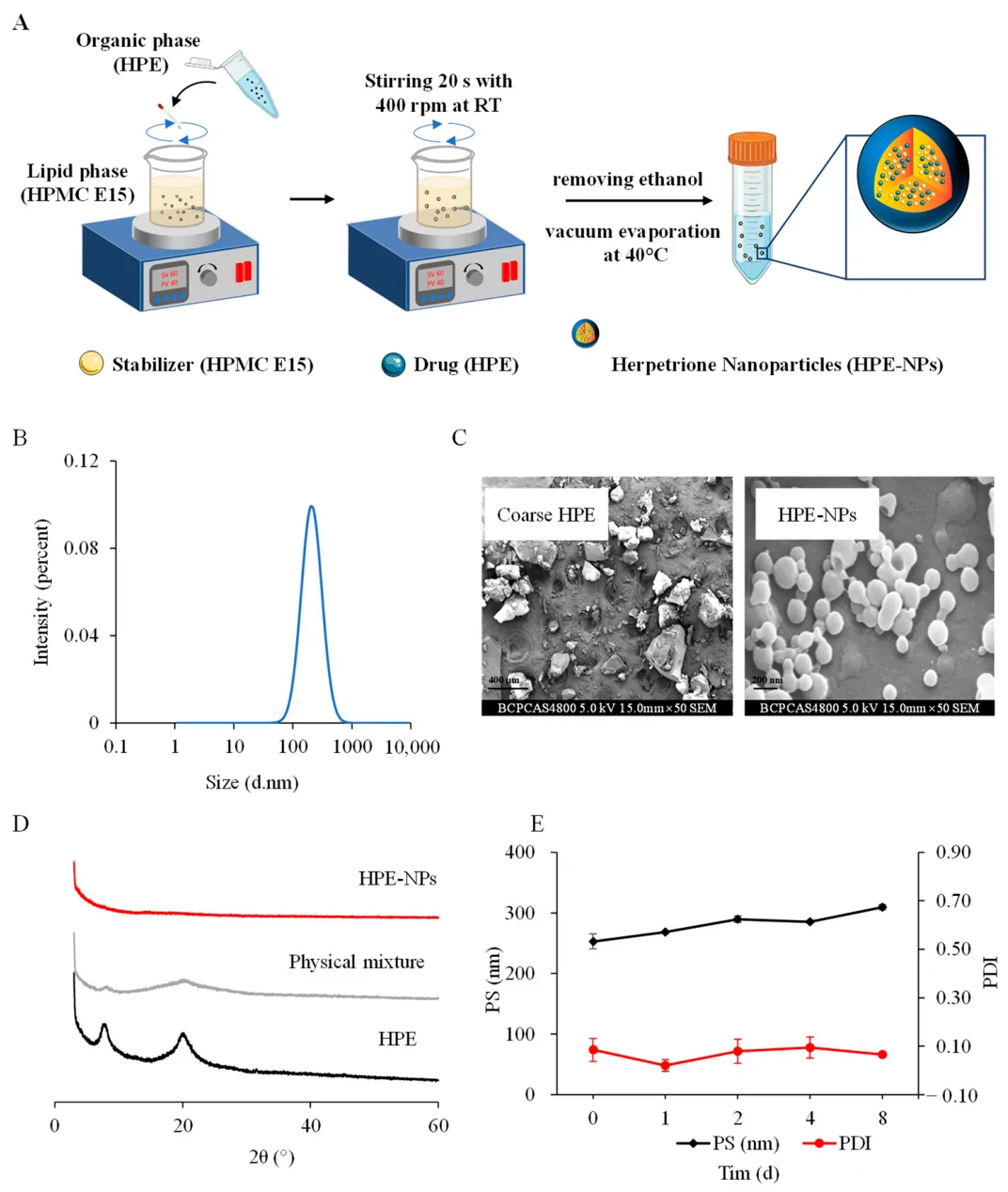
Preparation and physicochemical characterization of HPE-NPs. (**A**) Schematic workflow for sample preparation; (**B**) Particle size distribution; (**C**) SEM images of raw HPE and HPE-NPs; (**D**) XRD patterns of raw HPE, physical mixture, and HPE-NPs; (**E**) EE and LC values of HPE-NPs.

The schematic way of the preparation of HPE-NPs is presented in Figure 6A. The HPE-NPs exhibited uniform liquid appearance, with particles displaying a consistent spherical morphology and an average particle size of (274.79 ± 8.54) nm, the PDI was measured at 0.13 ± 0.05 (Figure 6B). The zeta potential was determined to be (-55.42 ± 2.04) mV. Notably, the HPE-NPs demonstrated a high DL content of 80.88 ± 0.63% and an EE of 80.01 ± 0.32%.

#### 3.8.2. Morphology

SEM images revealed substantial morphological differences between raw HPE and HPE-NPs (Figure 6C). While raw HPE appeared as irregularly shape, the HPE-NPs exhibited a uniform spherical morphology with smooth surfaces and no visible aggregation. The particle size observed through SEM was consistent with the measurements obtained via DLS.

#### 3.8.3. XRD Patterns

The XRD patterns for raw HPE, physical mixture (HPE + HPMC E15), and HPE-NPs are depicted in (Figure 6D). the Across the measured 2θ range, none of the samples displayed sharp or characteristic diffraction peaks. Instead, broad diffuse halos were observed at around 8.1° and 20.1°, the intensity of its NPs has decreased, suggesting that both raw HPE and HPE-NPs exist in an amorphous state. This is consistent with what is widely reported in the literature [19,20,21].

#### 3.8.4. Stability Evaluation

The short-term physical stability of HPE-NPs was evaluated by monitoring particle size and PDI during storage at 4 °C for 7 days. No significant change was observed over this period (Figure 6E), indicating acceptable stability for routine laboratory use and in vitro characterization.

#### 3.8.5. Solubility Improvement and Dissolution Kinetics

**Figure 7:**
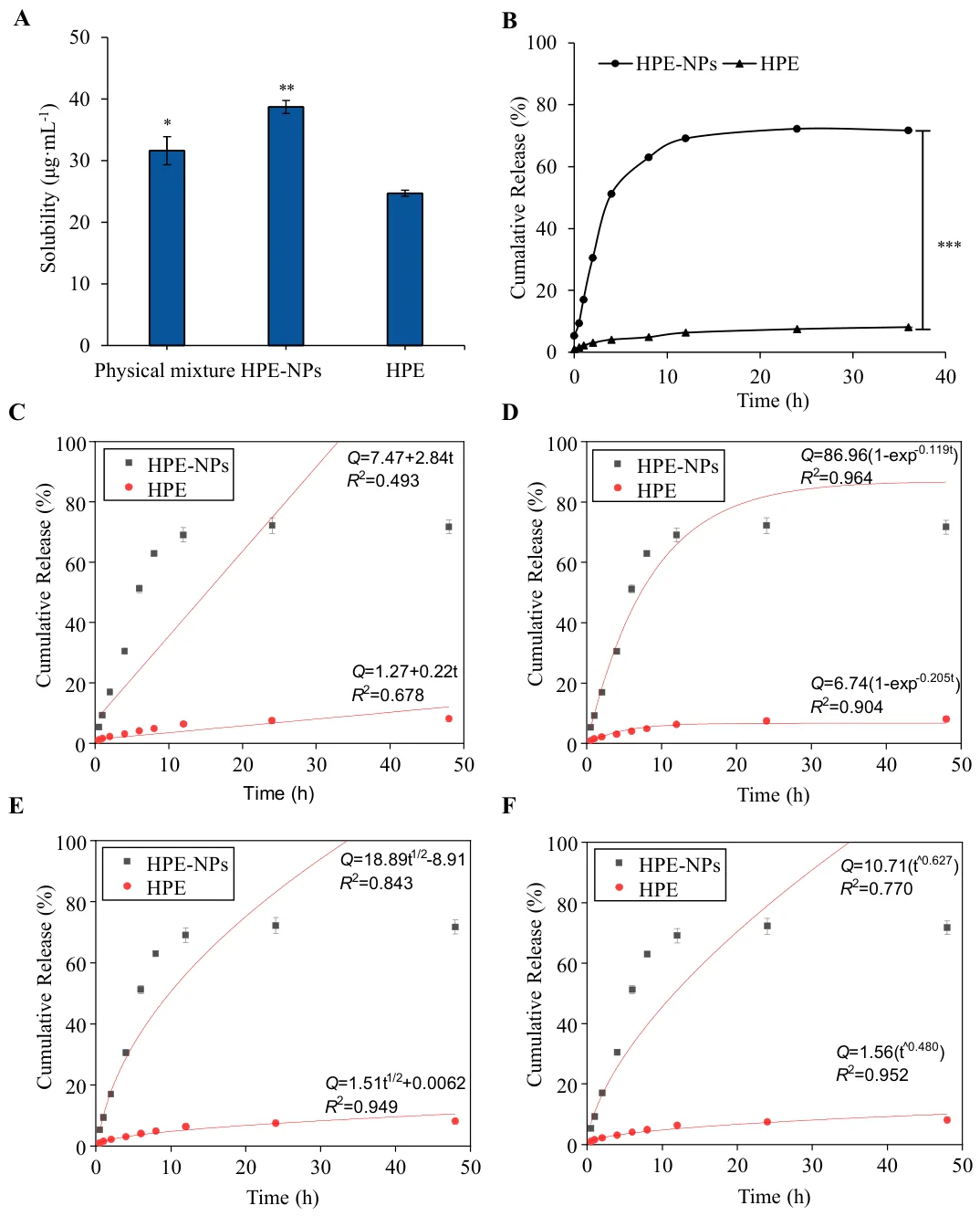
Solubility and in vitro drug release kinetics of HPE and HPE-NPs. (**A**) Equilibrium solubility. (**B**) Cumulative release profiles of HPE and HPE-NPs in PBS. (**C**) Fitting curve of release data to zero-order kinetic model, (**D**) first-order kinetics, (**E**) Higuchi model, (**F**) Ritger-Peppas model. Data are presented as mean ± SD (*n* = 3). Statistical significance of differences between formulations (vs. HPE): * *p* < 0.05, ** *p* < 0.01, *** *p* < 0.001.

The equilibrium solubility of HPE-NPs in water was found to be (38.68 ± 1.07) μg·mL^−1^, which is 1.5 times greater than that of raw HPE (24.68 ± 0.47) μg·mL^−1^) and 1.2-fold higher than that of the physical mixture (31.58 ± 2.26 μg·mL^−1^) (Figure 7A). This enhancement is primarily attributed to the increased surface area from the particle size reduction and the improved wettability provided by HPMC E15.

#### 3.8.6. In Vitro Dissolution

The in vitro dissolution profile is shown in Figure 7B for the HPE-NPs and raw drug. HPE showed a 6% accumulative release after 24 h, with a slower release thereafter. In contrast, HPE-NPs displayed a rapid initial burst release followed by a sustained phase, reaching approximately 72% cumulative release at 48 h. Compared to the pure drug, the HPE-NPs had a significantly higher release rate and degree. The release kinetics were analyzed using various models, as shown in Figure 7C–F. The raw HPE release kinetics fit the Ritger-Peppas model (*R*^2^ = 0.952), while HPE-NPs followed first-order kinetics (*R*^2^ = 0.964), indicating a shift from diffusion-controlled to concentration gradient-controlled release after nanocrystal formulation [59].

The nanoparticle formulation significantly enhanced both the solubility and dissolution rate of HPE. Nevertheless, this improvement does not alter the BCS classification of HPE, as the absolute solubility remains low. The primary value of the formulation lies in accelerating dissolution and maintaining supersaturation, rather than overcoming intrinsic low solubility. It should also be noted that the dissolution medium used does not fully reflect gastrointestinal physiology, which lacks key physiological components (e.g., bile salts, phospholipids, and variable pH gradients). Therefore, future studies employing biorelevant media, such as fasted-state simulated intestinal fluid (FaSSIF) or fed-state simulated intestinal fluid (FeSSIF), would be warranted to better predict in vivo dissolution behavior. Crucially, the extent to which these in vitro dissolution advantages translate into improved oral bioavailability remains unconfirmed, owing to the absence of in vivo pharmacokinetic data. Definitive validation requires prospective in vivo studies, including plasma concentration–time profiling following oral administration and the determination of absolute oral bioavailability, to rigorously assess the formulation’s therapeutic relevance and to determine whether the P-gp-mediated efflux barrier observed in vitro is mitigated under physiological conditions.

## 4. Conclusions

In summary, this work presents a case study demonstrating how integrated in silico, in vitro, and in situ approaches can guide the BCS classification and formulation design of natural products with unknown clinical profiles. Using HPE as a model, we identified it as a BCS class II compound, characterized by low solubility and high effective permeability. Notably, the Caco-2 bidirectional transport study revealed a high efflux ratio, and molecular docking confirmed strong binding of HPE to the P-gp substrate-binding pocket, collectively suggesting that HPE is likely a substrate of P-gp. Guided by this profile, a nanoparticle formulation was rationally developed using antisolvent precipitation with HPMC E15 as a stabilizer. The NP formulation markedly enhanced solubility, achieving a 1.5-fold increase in equilibrium solubility and improving the 48 h cumulative release from 6% to 72%. Collectively, this work provides a reference for BCS-guided formulation strategies for natural products. Nevertheless, the translational relevance of these findings remains to be confirmed in human studies, and the specific transporters involved in HPE absorption require further elucidation.

## Figures and Tables

**Table 1:** Physicochemical and biopharmaceutical predicted properties for HPE.

Parameter	Parameter	ALOGPS 2.1	ADMET lab 2.0	Swiss ADME	Average
Physicochemical	MW	-	552.20	552.58	-
	log *P*	2.94	2.43	2.78	2.716
	log *D*	-	2.34	-	-
	TPSA	-	144.14	144.15	-
	HBD	-	4	4	-
	HBA	-	10	10	-
	RB	-	9	9	-
	log *C*_s_	−4.89	−4.46	−4.60	−4.65
	*C*_s_	7.12	19.25	13.88	13.42
	*D*_0_	16.86	6.23	8.65	9.7
Absorption	Permeability	-	−5.32	-	-
	HIA	-	59.90%	Low	-
	P-gp substrate	-	No (0.203)	No	-

MW: molecular weight; log *P*: the n-octanol/water partition coefficient; log *D*: the n-octanol/water distribution coefficient; TPSA: topological polar surface area; HBD: hydrogen bond donor; HBA: hydrogen bond acceptor; RB: rotatable bond; log *C*_s_: represents the aqueous solubilities of HPE; *D*_0_: dose number; and HIA: human intestinal absorption.

**Table 2:** Predicted human intestinal permeability (*P*_eff, human_) and fraction dose absorbed (*F_a_*) based on rat jejunal intestinal permeability (*P*_eff, rat_) using established correlation models.

Compound	Herpetrione
*P*_eff, rat_ (×10^−5^ cm·s^−1^)	5.10
*P*_eff, human_ (×10^−5^ cm·s^−1^) [54]	18.67
Human *F_a_* (%) [54]	98.64
*P*_eff, human_ (×10^−5^ cm·s^−1^) [55]	26.32
Human *F_a_* (%) [55]	85.94

**Table 3:** Docking score results of particular ligands.

Target	Ligand	Binding Energy (Kcal·mol^−1^)
P-gp	Herpetrione	−8.998
	Quercetin	−8.686
	Original ligand	−7.652

## Data Availability

The authors state that the data supporting the conclusions of this study are available within the paper. All study data can be obtained from the corresponding author upon reasonable request.
